# Early ambulation after transfemoral diagnostic cerebral angiography: a pilot study

**DOI:** 10.1186/s12883-022-02561-0

**Published:** 2022-01-24

**Authors:** Zaid Aljuboori, Jessica Eaton, Kate Carroll, Michael Levitt, Louis Kim

**Affiliations:** Department of Neurosurgery, University of Washington School of Medicine, 325 Ninth Avenue, Box 359924, Seattle, WA 98104 USA

**Keywords:** Hematoma, Angiography, Transfemoral, Complications, Compression

## Abstract

**Background:**

A significant proportion of transfemoral cerebral angiography complications are related to the access site, with no clear consensus concerning the optimal closure technique. In this study, we examined the usefulness of a shortened closure protocol for transfemoral diagnostic cerebral angiography.

**Methods:**

We performed a retrospective review of patients who underwent transfemoral (4Fr sheath) diagnostic cerebral angiography procedures at our institution. We included patients > 18 years old who underwent the shortened closure protocol to achieve hemostasis at the access site. The shortened protocol entailed the use of nonocclusive manual compression for 15 min followed by 2 h of bed rest, with additional 10–15 min of compression for new hematoma. We collected and analyzed the patients’ demographics, use of antiplatelet and anticoagulation medications, sheath size, and others.

**Results:**

The study cohort comprised 119 patients with a mean age was 54 years with (88%) females. Forty-one patients (34%) were on antiplatelet medications, with 12 (10%) on dual antiplatelet therapy (DAPT). Four patients (3%) (two on DAPT, one on Aspirin alone, and one was not on any antiplatelet medication) had access site hematoma that required additional compression. Subgroup analysis showed that within the DAPT, Aspirin alone, and no antiplatelet medications groups, (17%), (3%), and (1%) of patients developed access site hematoma, respectively.

**Conclusion:**

This pilot study demonstrates that our closure protocol for transfemoral angiograms is safe and effective. There was a trend toward higher access-site complications in patients on DAPT. Further studies are required to expand on and validate our results.

## Background

Cerebral angiography is a diagnostic procedure to assess cerebral vasculature. It’s commonly performed using a transfemoral approach [1]. A wide range of complications have been connected to the transfemoral approach, with a significant proportion related to the access site (e.g., groin hematoma, pseudoaneurysm, arteriovenous fistula, etc.) [1–4]. There are different closure protocols with no clear consensus regarding the optimal technique [1–6]. In this study, we assessed the efficacy and safety of a shortened closure protocol for transfemoral diagnostic cerebral angiography.

## Methods

We performed a retrospective review of prospectively collected data concerning a shortened closure protocol for transfemoral diagnostic cerebral angiography procedures performed at the University of Washington between October 2020 and July 2021. All methods were carried out in accordance with relevant guidelines and regulations. No experimental protocols were used.

The shortened protocol involved 15 min of nonocclusive manual compression to the arterial access site followed by 2 h of bed rest, with additional 10–15 min of compression if hematoma formation occurred. We included patients > 18 years old who underwent a transfemoral diagnostic cerebral angiography using a 4-french femoral sheath and underwent the shortened closure protocol to obtain hemostasis at the access site. The primary outcome was the rate of access-site hematoma. We collected the patients’ demographics, comorbidities, use of antiplatelet and anticoagulation medications, femoral sheath size, and others. Also, we grouped the patients based on the use of antiplatelet agents into DAPT, single agent, and no antiplatelet agent. We calculated the comorbidity score of the patients using the Charlson-Deyo scoring system [7]. We calculated percentages, means, and standard deviation (SD). ANOVA and Chi-squared tests were used to compare the means and categorical outcomes among the groups, respectively. A significance level of *p* < 0.05 was used and the statistical analysis was done with Stata version 13 (StataCorp, College Station, TX).

## Results

We included 119 patients who underwent a transfemoral diagnostic cerebral angiogram using a 4-French femoral sheath. In all patients, access was obtained under ultrasound guidance. The mean age was 54 years with (88%) females. Forty-one patients (34%) were on antiplatelet medications, with 12 (10%) on dual antiplatelet therapy (DAPT). Intravenous heparin was not used during any of the procedures and none of the patients was on oral anticoagulation medications. Of all patients, (27%), (31%), (16%), and (26%) had Charleson-Deyo comorbidity score of 0,1, 2, and =  > 3, respectively. Four patients (3%) (two on DAPT, one on Aspirin alone, and one was not on any antiplatelet medication) had access-site bleeding complications after 15 min of manual compression. In all four patients, the bleeding can be characterized as a small leaking of blood from the groin puncture site that required additional compression to achieve hemostasis with no further intervention. Also, one of these patients presented with delayed hematoma due to a small pseudoaneurysm, which was treated conservatively. Also, the analysis of individual groups showed that in patients on single or no antiplatelet medications, the rate of access site bleeding complications was (1.7%) hematoma [Tables 1 & 2]. Moreover, within the DAPT, single antiplatelet agent, and no antiplatelet medications groups, (17%, *N* = 2), (3%, *N* = 1), and (1%, *N* = 2) of patients developed access site hematoma, respectively. Comparative analysis revealed no significant difference in age (*p* = 0.1) and comorbidity score (*p* = 0.1) among the three groups. Correlation analysis revealed a significant correlation between the use of antiplatelet agents and the occurrence of access site hematoma (OR = 4, 95% CI:1.1–14.7, *p* = 0.03). Moreover, there were no occurrences of major complications (e.g., retroperitoneal hematoma), arteriovenous fistula, or femoral artery dissection.

**Table 1:** Demographics of the study population

**Variable**			**N (119)**	**Percentage**
**Age (years)**	**Range**	19–86		
	**Avg**	54		
**Gender**	**Male**		14	12%
	**Female**		105	88%
**Antiplatelets**	**DAPT**		12	10%
	**ASA or Plavix**		29	24%
**CCI**	**0**		32	27%
	**1**		37	31%
	**2**		19	16%
	** = > 3**		31	26%

*Avg* average, *ASA* Aspirin, *CCI* Charlson comorbidity index, *DAPT* dual antiplatelets therapy

**Table 2:** Details of access-site complications

**Variable**		**N**	**Percentage**
**Groin hematoma**	**DAPT group**	2	17%
	**ASA or Plavix group**	1	3%
	**No antiplatelets group**	1	1%
	**Total**	4	3%
**Femoral artery pseudoaneurysm**		1	0.8%

*ASA* Aspirin, *DAPT* dual antiplatelets therapy

## Discussion

In diagnostic cerebral angiography, both the femoral and radial arteries are commonly used to access the arterial system [1, 6]. For the transfemoral approach, the access site complications include hematoma (0.5–1.7%), pseudoaneurysm and arteriovenous fistula (0.1–0.6%), and infection (0–1%) [1–4]. Several methods are available to obtain access site hemostasis such as manual nonocclusive compression, external compression devices, and vascular closure devices [1–4, 8]. Historically, hemostasis at the access site was obtained through pressure dressing followed by six hours of bed rest, with a recent trend toward the use of manual compression with early mobilization [2, 4, 5].

Our results showed that the overall rate of groin hematoma was (3%), which is higher than other closure techniques [1–4]. Analysis among the groups showed no significant difference in age and comorbidity score. Moreover, the rate of access-site hematoma was (1%), (3%), and (17%) for patients who were on no antiplatelet agents, single agent, and DAPT, respectively. Also, there was a significant association between the use of antiplatelet agents and the development of access site hematoma (OR = 4). Tonetti et al.[4] reported a (1.1%) rate of access site hematoma after 20 min of manual compression in patients who underwent transfemoral angiography and were on one or more antiplatelet agents. Also, their results revealed a rate of (0.8%) in patients not on antiplatelet agents. The higher rate in our study could be due to the difference in the sample size (529 vs 119 patients in our study) or the difference in the protocol (20 vs 15 min of manual compression in our study).

Our results indicate that patients on DAPT are more likely to develop access site hematoma and may require a longer duration of manual compression (e.g., =  > 20 min) and or a longer duration of bed rest (e.g., 4 h). Moreover, our protocol offers several potential advantages such as less patient discomfort (e.g., back pain) due to a shorter duration of bed rest. Also, it can lead to a quicker turnover of cases, which can minimize the effects of crowding. Furthermore, our protocol offers economic advantage as it obviates the cost associated with using a closure device. Moreover, vascular closure devices has a failure rate of (2–5%) and can result in a rare but serious complications such as acute limb ischemia [1, 3, 8, 9].

### Limitations

This is a single-institutional study, which can lead to selection bias. Also, our study population included patients who underwent diagnostic cerebral angiography only. If applied to patients who undergo endovascular intervention, our protocol might result in a higher access site complications since there is a potential need for intraoperative antiplatelet or anticoagulation medications. Moreover, failure of reporting of small hematomas by the patients could have affected the results of this study.

## Conclusion

Transfemoral cerebral angiography is a common procedure with no clear consensus regarding the optimal closure technique. Our results show that the shortened closure protocol is effective with a good safety profile. Also, we found that patients on dual antiplatelet therapy at higher risk for access site complications.

## Data Availability

The datasets used and/or analyzed during the current study are available from the corresponding author on reasonable request.
